# Long-Term Effects of Nilvadipine against Progression of the Central Visual Field Defect in Retinitis Pigmentosa: An Extended Study

**DOI:** 10.1155/2013/585729

**Published:** 2013-11-12

**Authors:** Mitsuru Nakazawa, Yukihiko Suzuki, Tadashi Ito, Tomomi Metoki, Takashi Kudo, Hiroshi Ohguro

**Affiliations:** ^1^Department of Ophthalmology, Hirosaki University Graduate School of Medicine, 5 Zaifu-cho, Hirosaki 036-8562, Japan; ^2^Department of Ophthalmology, Sapporo Medical University, S1W17, Chuo-ku, Sapporo 060-8556, Japan

## Abstract

*Purpose*. To assess the long-term effects of nilvadipine on the progression of central visual field defect in retinitis pigmentosa (RP). *Methods*. Patients with RP were randomly divided into a treated group receiving oral nilvadipine and a control group. Progression of RP was evaluated with MD slope and the average sensitivity of the central 2° (ΔCENT4). *Results*. The mean MD slopes were −0.55/−0.39 (right/left eyes, *n* = 19) dB/year in the treated group and −1.37/−1.15 (right/left eyes, *n* = 22) dB/year in the control group (*P* = 0.016/0.050, resp.). In both eyes, however, no statistical difference was observed between the two groups for the ΔCENT4 values. *Conclusion*. Although we confirmed that nilvadipine significantly retarded the progression of the average of MD value defects in the central 10°, it was not specific for the central 2° of the visual field in RP.

## 1. Introduction

 Retinitis pigmentosa (RP) denotes a heterogeneous group of hereditary retinal degenerations and comprises one of leading causes of visual handicaps. Investigation of the molecular genetic background of RP has revealed that more than 100 different genes may be involved, including syndromic RP (Ret-Net: Disease Table. http://www.sph.uth.tmc.edu/retnet/disease.htm). In addition to variation in the genotypes, the RP phenotypes in terms of progression and severity are also known to vary. As in general the photoreceptor degeneration seen in RP patients starts with rod degeneration, these patients also subjectively complain of night blindness and eventual peripheral and/or midperipheral visual field constriction during the relatively early stage of the disease. In more advanced stages, however, the central vision is also reduced, as the survival of the cone photoreceptors appears to be dependent on the rods [1]. Therefore, when many of these RP patients visit ophthalmologists, their central visual field has already been disturbed to some extent. Thus, serially monitoring the central visual field is important for patients with RP so that clinicians can both determine the level of the visual handicaps and be able to speculate on the actual progression of the disease. As for the monitoring of the visual sensitivity of RP patients, Iijima recently reported that the mean deviation (MD) of the 10-2 program of the Swedish Interactive Threshold Algorithm (SITA) for the Humphrey Visual Field Analyzer (HVFA, Humphrey Instruments, San Leandro, CA) might be the best variable for monitoring the progression of visual dysfunction in general in RP [2]. Iijima's study also found that the average sensitivity for the central 4 points (CENT4) was correlated with the central visual acuity and proved better than the MD value for monitoring the advanced stages of RP. Furthermore, this previous study additionally demonstrated the CENT4 values are more correlated to the visual progression of RP than the central visual acuity.

After our previous studies demonstrated the protective effects of nilvadipine against photoreceptor degeneration in 3 different kinds of RP animal models [3–6], we began a prospective, randomized, nonmasked clinical trial that examined the effect of nilvadipine on the central visual field defect in RP (UMIN000007895) [7]. After our previous report [7], we extended the study to evaluate the long-term safety and the further effects of nilvadipine on RP and serially monitored the visual sensitivities measured by the 10-2 program of HVFA. In this current clinical trial, we compared the time courses for the visual sensitivities determined by not only the MD values in the central 10° but also those in the CENT4 of the visual field, as the CENT4 values were not analyzed in our previous study [6]. In the current extended study, because visual acuity is one of the most important visual functions besides the visual field in RP patients, we further analyzed the effects of nilvadipine on the central visual sensitivity (CENT4) as well as the averaged visual sensitivity (MD) in the central 10° of the visual field. Furthermore, we statistically analyzed our data using a multivariate regression analysis in order to determine which factors significantly affected the prognosis of the visual functions in RP patients. We report herein the results of this extended study.

## 2. Subjects and Methods

 This study (UMIN000007895) was carried out in the Department of Ophthalmology at Hirosaki University Hospital. All study protocols were approved by the Institutional Committee of Ethics for Medical Research and complied with the ethical standards of the 2000 Declaration of Helsinki. Informed consent was obtained from all patients.

 A clinical diagnosis of RP was determined based on the following criteria: (1) fundus examination showing typical arteriolar attenuation and bilateral mottled appearance of fundus color with or without retinal pigmentation; (2) rod-cone degeneration type with rod-predominant degeneration shown on electroretinography (ERG) and/or subjective symptoms; and (3) constriction of visual field defects evaluated by either a Goldmann perimetry or an HVFA (30-2 or 10-2 SITA Fast Programs).

From among patients with RP seen in the outpatient retina clinic of the Department of Ophthalmology at Hirosaki University Hospital from July 2001 to September 2009, patients eligible for inclusion were invited to participate in the present study. Inclusion criteria were age between 20 and 75 years; residual central visual field better than −35.0 decibels (dB) as evaluated by the 10-2 SITA Fast Program; reliable results of the visual field examination, that is, visual field data with fixation loss scores of ≤20% and false-negative error ≤33%; and best-corrected visual acuity better than 0.1. Exclusion criteria included intraocular surgery during the observation period of each patient; presence of cataracts or other media opacities that would disturb visual field examination; use of other calcium channel blockers to treat systemic illnesses; retinal degenerations secondary to inflammation; or the presence of ocular diseases other than RP.

In line with our previously described method [6], patients were randomly divided into a treated group and a control group using a simple randomization. However, all patients with a systolic blood pressure <110 mmHg prior to participation were automatically enrolled into the control group. Briefly, patients in the treated group were prescribed 4 mg/day of oral nilvadipine (2 mg capsule, twice daily; Astellas Pharmaceutical, Tokyo, Japan). Patients in the control group were periodically followed either without any prescription or with tocopherol nicotinate (100 mg capsule, 3 times/day; Eisai, Tokyo, Japan) or helenien (xanthophyll dipalmitate, 5 mg tablet, 3 times/day; Bayer Health Care, Osaka, Japan) according to the wishes of the patients. On deciding which prescription the patients in the control group would be given, these patients were provided with a complete explanation of the possible effects and limitations of tocopherol and helenien on the prognosis of RP. The original protocols in the patients who had taken part and completed the previous study [6] were not suspended for as long as possible while these patients received further extended followups. Using the methods reported by Hirakawa et al. [8], two ophthalmic technicians who were completely blinded to the group allocations performed the visual function measurements using the 10-2 SITA Fast Program of the HVFA every 6 months. The study was completed in September 2012 and all follow-up data were analyzed in October 2012. Three different methods were used to analyze the long-term effects of nilvadipine on the progression of central visual field defects in RP. First, distributions of the regression coefficients were calculated from time courses of the MD values of the 10-2 program (dB/year, MD slope) during the total observation period for each group (final MD slope), with the groups then compared (Analysis 1). Second, distributions of the regression coefficients were calculated from the time courses of the average sensitivity of the 4 central tests points (CENT4) of the 10-2 program (dB/year, ΔCENT4), with the values then compared between the groups (Analysis 2). In addition, regression coefficients (dB/year) and Spearman's correlation coefficients were determined between both of the eyes examined in Analyses 1 and 2, with all calculations performed using SPSS version 19 software (Statistical Package for the Social Sciences, SPSS, Chicago, IL). Third, patients' ages at enrollment, ages at the initial symptom or diagnosis of RP, observation periods, initial MD values, gender, and whether or not patients underwent treatment with nilvadipine were examined as independent variables using SPSS so that a step-wise multivariate regression analysis could be performed to determine which factors most likely affected the final MD slope or ΔCENT4 values (dependent variable) (Analysis 3). Statistical differences were examined by the Mann-Whitney *U*-test in Analyses 1 and 2 using SPSS. Values of *P* ≤ 0.05 were considered statistically significant.

## 3. Results

 All of the 41 patients initially enrolled (treated group, *n* = 19; control group, *n* = 22) in the present study completed followups of at least 30 months. Averaged observation periods were 72.32 ± 24.29 months for the treated group and 65.73 ± 22.26 months for the control group (mean ± standard deviation). No complications due to the treatment such as skin eruption, headache, dizziness, or other subjective complaints related with the autonomic nervous system were seen throughout any of the followups. No patients showed systemic hypotension <110 mmHg. Of the 22 patients in the control group, 5 patients requested prescription of tocopherol nicotinate, 9 patients requested helenien, and 8 patients did not request any medication during their followups. Background and clinical data for the 41 participants are summarized in Table 1. No significant differences were noted between the groups for the patient's ages at enrollment, initial symptoms or diagnosis, gender, mean total observation periods, and severity of disease evaluated by MD values and CENT4 in both eyes at the time of enrollment (Table 1). Significant correlations between the right and left eyes were noted for the MD values and CENT4 (Table 1). 

**Figure 1:**
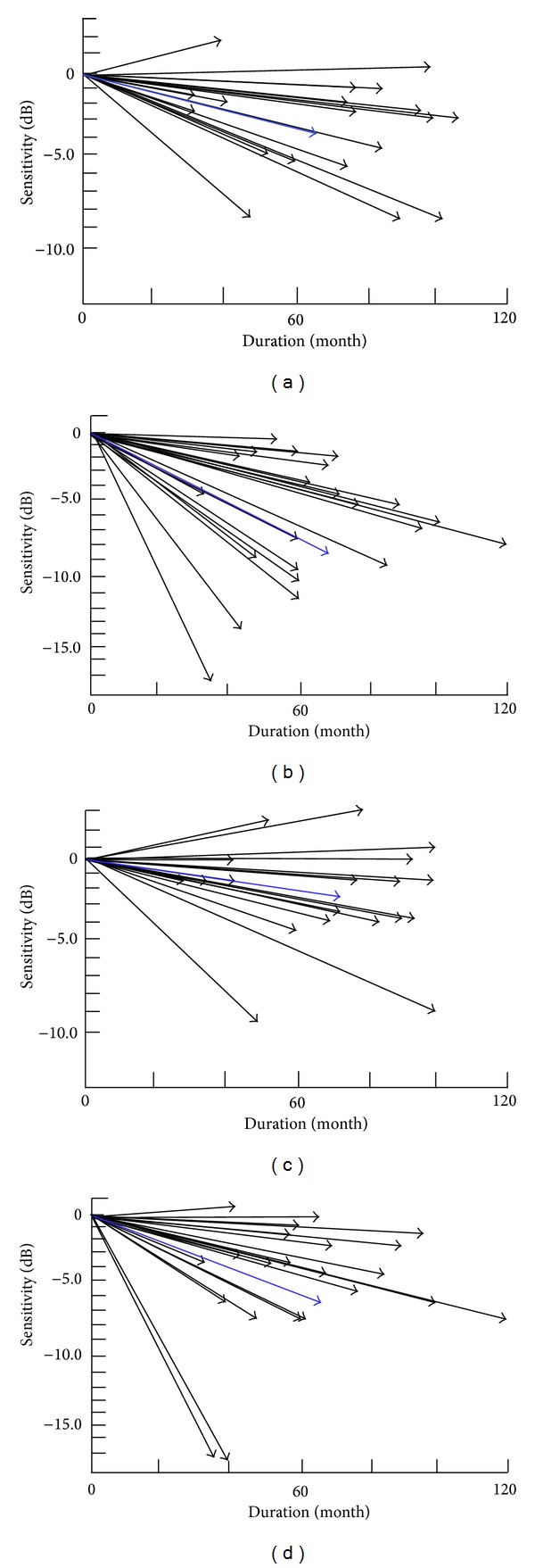
Distribution of the MD slope for each participant during the observation periods. (a) Right eyes of the treatment group; (b) Right eyes of the control group; (c) Left eyes of the treatment group; (d) Left eyes of the control group. Black arrows indicate each patient's MD slope; blue arrows indicate the average value for each group.

Mean (±standard error of the mean, SEM) of the MD slope during the entire observation period for each patient (Analysis 1) is summarized in Table 2. Mann-Whitney *U*-test results showed there was a significant difference between the groups (*P* = 0.016 in the right eye and *P* = 0.050 in the left eye; Table 2). Spearman's correlation coefficients between both eyes were 0.715 (*P* = 0.001) in the treated group and 0.836 (*P* < 0.001) in the control group. The MD slopes for all of the participants are presented in Figure 1. Mean (±SEM) of ΔCENT4 during the entire observation period for the individual patients (Analysis 2) is shown in Table 3. No significant differences were noted between the groups (*P* = 0.296 in the right eye and *P* = 0.100 in the left eye, Mann-Whitney *U*-test; Table 3). Results of multivariate regression analysis are summarized in Table 4. As for MD slope values, statistically significant partial correlation coefficients were found by the step-wise multivariate regression analysis with 0.715 (*P* = 0.036) in the right eye and 0.779 (*P* = 0.042) in the left eye for the nilvadipine treatment, while 0.015 (*P* = 0.047) was determined in the right eye for the observation periods. None of the other factors analyzed as independent values were found to be statistically significant. As for ΔCENT4 values, no statistically significant relationship was found.

## 4. Discussion

Irrespective of the involvement of the calcium channel-blocking actions, the increased blood flow, or both, the present study does indicate that nilvadipine can slow the progression of the central visual field defect in RP for a long-term period. Assessment by the 10-2 program in Analysis 1 estimated that the annual protection induced by 4 mg/day of oral nilvadipine was, on average, 0.82 (right eye) to 0.77 (left eye) dB/year over that found in the control patients, both of which were statistically significant (*P* = 0.016 and 0.050, resp.). Results of multivariate regression analysis indicated that nilvadipine treatment had the most significant effect on the difference in the MD slope bilaterally between the two groups. Although effects of nilvadipine were only found in the MD slope and not in the CENT4, these results also support the finding that nilvadipine is safe and has a beneficial effect on the progression of the visual field dysfunction rather than the central visual acuity of RP for a relatively long time.

Similar to the previous study by Hirakawa et al. [8], the present study also used the 10-2 SITA Fast Program of the HVFA to evaluate visual functions in patients with RP. Since vision in most of these patients was generally constricted to the central visual field, it was better to perform quantitative assessments using MD values from the 10-2 program versus those from the 30-2 program. Moreover, when ERG has been used to evaluate photoreceptor sensitivity, exponential decreases have been reported to occur as the RP progresses [9]. Since MD values are logarithmically converted from the retinal sensitivity, the decrease of the MD values can be considered to be linear. Thus, as has been previously reported, the linear MD slope (dB/year) can be regarded as an indicator of the speed of progression for RP [6]. Since Iijima recently reported that there was a high correlation between visual acuity and retinal sensitivity in the central 2° in the 10-2 program [2], our study further evaluated cone specific sensitivity by taking the CENT4 into account.

Our present study demonstrated that orally administrated nilvadipine (4 mg) was effective in retarding, on average, the progression of the central visual field defects in patients with RP when the progression was evaluated by the MD slope. Conversely, nilvadipine exhibited no statistically significant effects on the progression of CENT4 (ΔCENT4) in patients with RP, even though the averages of ΔCENT4 in the treatment group were slightly better than those in the control group. Although the mechanism for the discrepancy between MD slope and ΔCENT4 after nilvadipine treatment is currently unknown, it needs to be examined in a subsequent study. One possible explanation may be shown by the results from the 14 out of the 22 patients in the control group who were given either tocopherol nicotinate or helenien. Helenien is xanthophyll dipalmitate which is subsequently converted into lutein in the body after absorption. Both tocopherol and lutein have been known to have antioxidative effects and to have reportedly beneficial effects on RP [10, 11]. Because no patient in the treated group was given either tocopherol or helenien, their putative antioxidative effects in the control group might have masked the effect of nilvadipine on the central visual function in the treated group. If this is the case, in the future these antioxidative effects of tocopherol and lutein and the antiapoptotic effect of nilvadipine may be useful in providing synergistic effects to ameliorate progression of photoreceptor degeneration. 

Previously, only the calcium blockers nilvadipine and diltiazem have been examined for their effect on the progression of RP. However, the effect of diltiazem on photoreceptor protection remains controversial [12, 13]. We have previously shown that systemic administration of nilvadipine was effective for protecting photoreceptors from photoreceptor apoptosis in three RP models with different genetic backgrounds, such as the RCS rats [2, 3], rd1 mice [4], and heterozygous rd2 mice [5]. It has been speculated that one of main effects of nilvadipine may be the inhibition of the increase of intracellular calcium ions that leads to triggering the photoreceptor apoptosis [14]. The importance of calcium ions in apoptosis is known as it has been shown that calcium ions activate both caspases and calpains, both of which work as the main effectors in apoptosis are activated by calcium ions [15, 16]. 

 Our present results also suggest that phenotypic variability may also play a role in patients with RP. As seen in Figure 1, the distribution of the MD slopes of the patients with RP varied widely. Thus, the different effects seen for nilvadipine from patient to patient could depend on their different genetic background. Jin et al. recently demonstrated that rod photoreceptors induced from the RP patients' induced pluripotent stem (iPS) cells exhibited different sensitivities to vitamin E depending on the genetic defects [17]. Their study implies that in the future we will be able to check the efficacy of nilvadipine by examining the photoreceptors induced from each patient's own iPS cells. In addition, in this study we employed the patients' perimetric data simply because HVFA is the simplest, most convenient, and less invasive method for patients to perform among other methods including cone ERG [9], macular focal ERG [18], microperimetry [19], and optical coherent tomography. Further studies that examine the association between these factors and patients' prognoses will need to be undertaken.

## Figures and Tables

**Table 1:** Background clinical data for patients in the treated and control groups.

	Treated group	Control group	Statistical significance
Number of patients	19	22	
Gender (M/F)	9/10	10/12	
Age, years at enrollment	51.84 ± 10.94	48.41 ± 16.03	0.583
Median (range), years	52 (28–72)	49 (22–73)	0.168
Age, years at initial symptom or diagnosis	41.11 ± 1450	31.91 ± 19.47	0.105
Median (range), years	45 (10–66)	26 (6–72)	0.019*
Observation period, months	72.32 ± 24.29	65.73 ± 22.26	0.324
Median (range), months	78 (30–120)	78 (36–120)	0.087
Initial MD value, R, dB	−17.50 ± 10.82	−18.03 ± 6.21	0.896
Median (range), dB	−16.81 (−32.22~+0.69)	−17.88 (−29.92~−7.83)	0.647
Initial MD value, L, dB	−18.12 ± 11.34	−17.63 ± 6.70	0.834
Median (range), dB	−15.90 (−33.44~+0.24)	−16.57 (−27.72~−4.50)	0.647
Correlation between R and L	0.96	0.82	<0.001 (T, C)**
Initial sensitivity in CENT4, R, dB	24.67 ± 7.89	24.57 ± 6.02	0.935
Median (range), dB	24.75 (11.75–36.75)	23.75 (13.00–34.75)	0.342
Initial sensitivity in CENT4, L, dB	24.44 ± 7.54	23.40 ± 7.65	0.616
Median (range), dB	24.25 (14.25–36.75)	21.50 (12.00–34.75)	0.342
Correlation between R and L	0.80	0.63	<0.001 (T)** 0.002 (C)**

Data are expressed as means ± standard deviation.

M: male; F: female; R: right eye; L: left eye; T: treated group; C: control group.

Significance: *P≦0.05; **P < 0.01.

**Table 2:** Results of nilvadipine treatment on MD slope (Analysis 1).

	Treated group	Control group	Statistical significance
	(*n* = 19)	(*n* = 22)	*P* value
Averaged MD slope, R, dB	−0.55 ± 0.14	−1.37 ± 0.29	0.016*
Median (range), dB	−0.42 (−2.19~+0.06)	−0.81 (−5.94~−0.1)	0.016*
Averaged MD slope, L, dB	−0.39 ± 0.15	−1.15 ± 0.32	0.050*
Median (range), dB	−0.45 (−2.46~+0.60)	−0.76 (−5.82~+0.13)	0.168
Correlation between R and L	0.72	0.84	0.001 (T)** <0.001 (C)**

Data are expressed as means ± SEM.

R: right eye; L: left eye; T: treated group; C: control group.

Significance: *P≦0.05; **P < 0.01.

**Table 3:** Results of nilvadipine treatment on sensitivity of central 4 points (CENT4) (Analysis 2).

	Treated group	Control group	Statistical significance
	(*n* = 19)	(*n* = 22)	*P* value
Averaged ΔCENT4, R, dB/Y	−0.78 ± 0.25	−1.29 ± 0.39	0.296
Median (range), dB/Y	−0.67 (−3.60~+0.95)	−1.10 (−5.28~−1.85)	0.278
Averaged ΔCENT4, L, dB/Y	−0.51 ± 0.29	−1.75 ± 0.29	0.100
Median (range), dB/Y	−0.70 (−3.52~+2.65)	−1.12 (−13.47~+2.45)	0.087
Correlation between R and L	0.84	0.62	<0.001 (T)** 0.003 (C)**

Data are expressed as means ± SEM.

R: right eye; L: left eye; Y: year; T: treated group; C: control group. Significance: *P≦0.05; **P < 0.01.

**Table 4:** Results of step-wise multivariate regression analysis for factors independently contributing to final MD slope (Analysis 3).

Independent values	Right eyes	Left eyes
	*b* value (*P* value)	*b* value (*P* value)
Nilvadipine treatment	0.715 (0.036*)	0.779 (0.042*)
Observation period	0.015 (0.047*)	Excluded (0.113)
Patients' age at enrollment	Excluded (0.923)	Excluded (0.404)
Patients' age at initial symptoms or diagnosis	Excluded (0.903)	Excluded (0.254)
Gender	Excluded (0.622)	Excluded (0.174)
Initial MD values	Excluded (0.927)	Excluded (0.681)

Significance: *P < 0.05.
